# Three-Dimensional Planning and Surgical Correction of Bilateral Developmental Valgus Deformity of the Manus in a Dog

**DOI:** 10.3390/ani16182919

**Published:** 2026-09-17

**Authors:** Francesca P. Solari, Mu Young Kim, Federico R. Vilaplana Grosso, Adam H. Biedrzycki, Daniel D. Lewis

**Affiliations:** 1 Department of Small Animal Clinical Sciences, College of Veterinary Medicine, University of Florida, Gainesville, FL 32608, USA; 2 Department of Large Animal Clinical Sciences, College of Veterinary Medicine, University of Florida, Gainesville, FL 32608, USA

**Keywords:** metacarpal bones, angular deformity, three-dimensional printing, virtual surgical planning

## Abstract

A one-year-old, 48 kg, male neutered, Great Dane presented for bilateral thoracic limb lameness ascribed to an apparent developmental valgus deformity of both manus. Corrective radial-shaped osteotomies of the four weight-bearing metacarpal bones (II–V) were virtually planned based on CT scan data, and bone models of each corrected manus were three-dimensionally (3D)-printed, sterilized, and used intra-operatively to facilitate the procedure. Corrective radial-shaped osteotomies of metacarpals II–V were performed on the right and then left manus, nine weeks apart. In the right manus, metacarpal bones II, IV, and V were individually stabilized with bone plates and screws, while metacarpal bone III was stabilized with an intramedullary pin. Bone plates and screws were used to stabilize metacarpal bones II–V in the left manus. Postoperative alignment was deemed appropriate following both procedures. A caudally positioned splint was maintained for four weeks following each surgery, and all osteotomies obtained union within eight weeks of surgery. The dog was examined 79 weeks after the initial procedure and thoracic limb posture and gait were markedly improved. Virtual surgical planning (VSP) and printed bone models facilitated successful correction of this dog’s deformities.

## Simple Summary

This case report describes the use of virtual surgical planning (VSP) and three-dimensionally (3D) printed models for surgical correction of bilateral, presumably developmental, metacarpal bone deformities in a dog. The use of VSP and 3D-printed models of the corrected manus during surgery allowed effective correction of the deformity, resulting in a good clinical outcome in this dog. This surgical approach could also be considered for correction of metacarpal deformities secondary to fracture malunion.

## 1. Introduction

Developmental thoracic limb deformities occur relatively frequently in dogs, predominantly involving the antebrachium. Antebrachial deformities can develop due to genetic inheritance, physeal trauma, chondrodysplasia, fracture malunion, nutritional imbalances or deficiencies, or metabolic disorders [1,2]. Surgical correction of antebrachial deformities is often advocated to address joint incongruity and/or to improve functional limb alignment [3,4,5].

Metacarpal deformities have been reported in dogs secondary to fracture malunion [6,7], with malunion reportedly occurring in up to 13% of metacarpal fractures managed conservatively [6]. The authors, however, were unable to locate prior reports describing developmental deformity of one or both manus (with the manus comprising the carpus, metacarpus, and digits) without a history of prior trauma in small animals. The objectives of this case report were to describe presumed developmental bilateral valgus deformity of the manus in a one-year-old dog and to describe successful mechanical and anatomical realignment of both manus by performing radial-shaped osteotomies of the four weight-bearing metacarpal bones (II–V) facilitated by preoperative virtual surgical planning (VSP) and intraoperative utilization of three-dimensionally (3D)-printed models of each corrected manus.

## 2. Case Description

### 2.1. Signalment, History, and Clinical Findings

A one-year-old male neutered Great Dane weighing 48 kg was referred to the University of Florida Small Animal Orthopedic Surgery Service for evaluation of bilateral thoracic limb lameness ascribed to angular deformities. The owners first noticed alterations in the dog’s thoracic limb gait several months prior to presentation. The gait alterations were characterized by outward deviation of both forepaws, and the severity of lameness had progressed over the two months prior to presentation. The dog seemed reluctant to bear weight on the thoracic limbs and would intermittently and alternately hold one of the thoracic limbs off the ground when sitting or standing. The owners perceived that the abnormalities were more profound in the right thoracic limb. Additionally, the owners noted that the dog excessively groomed both forepaws.

**Figure 1:**
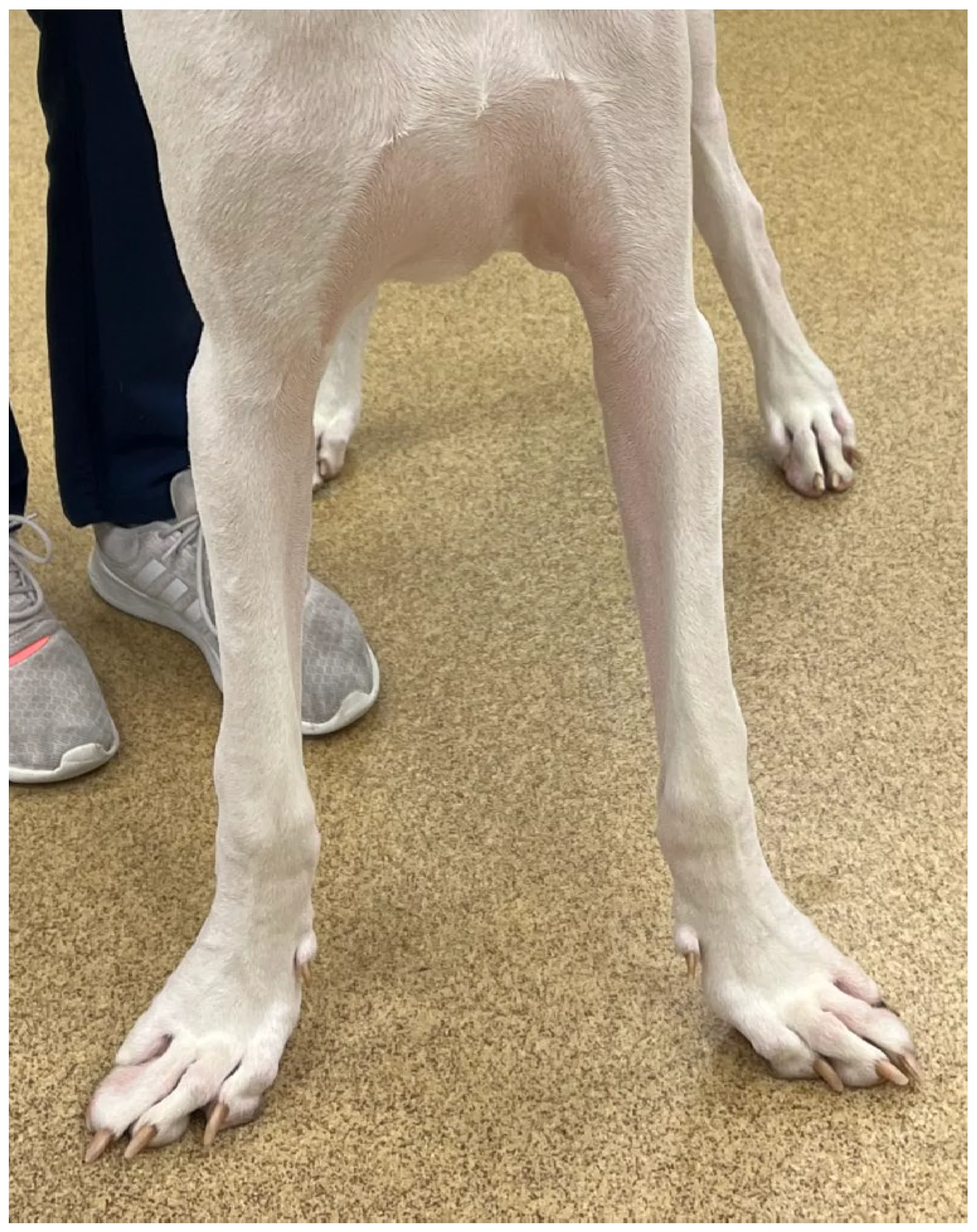
Representative image of the thoracic limbs of the dog on initial presentation showing bilateral valgus angulation of both manus, with the deformity primarily located distal to the carpi. When standing and while ambulating, the dog was predominately weight-bearing on the medial surface of digits II–IV.

On orthopedic examination, there was a symmetrical thoracic limb gait abnormality characterized by circumduction while advancing both thoracic limbs during the swing phase of the stride. There was bilateral valgus angulation of both manus, and the dog predominantly bore weight on the medial aspect of digits II–IV when walking and standing. The deformity appeared to be isolated to the manus without obvious visual deformity of either antebrachium (Figure 1). The dog reacted with an apparent mild pain response when digital pressure was applied to the dorsum of the right, but not the left, metacarpus. Range of motion in all joints of both thoracic limbs was normal, and pain was not elicited on manipulation of any joint in either thoracic limb. No neurologic abnormalities were noted.

### 2.2. Diagnostic Imaging

**Figure 2:**
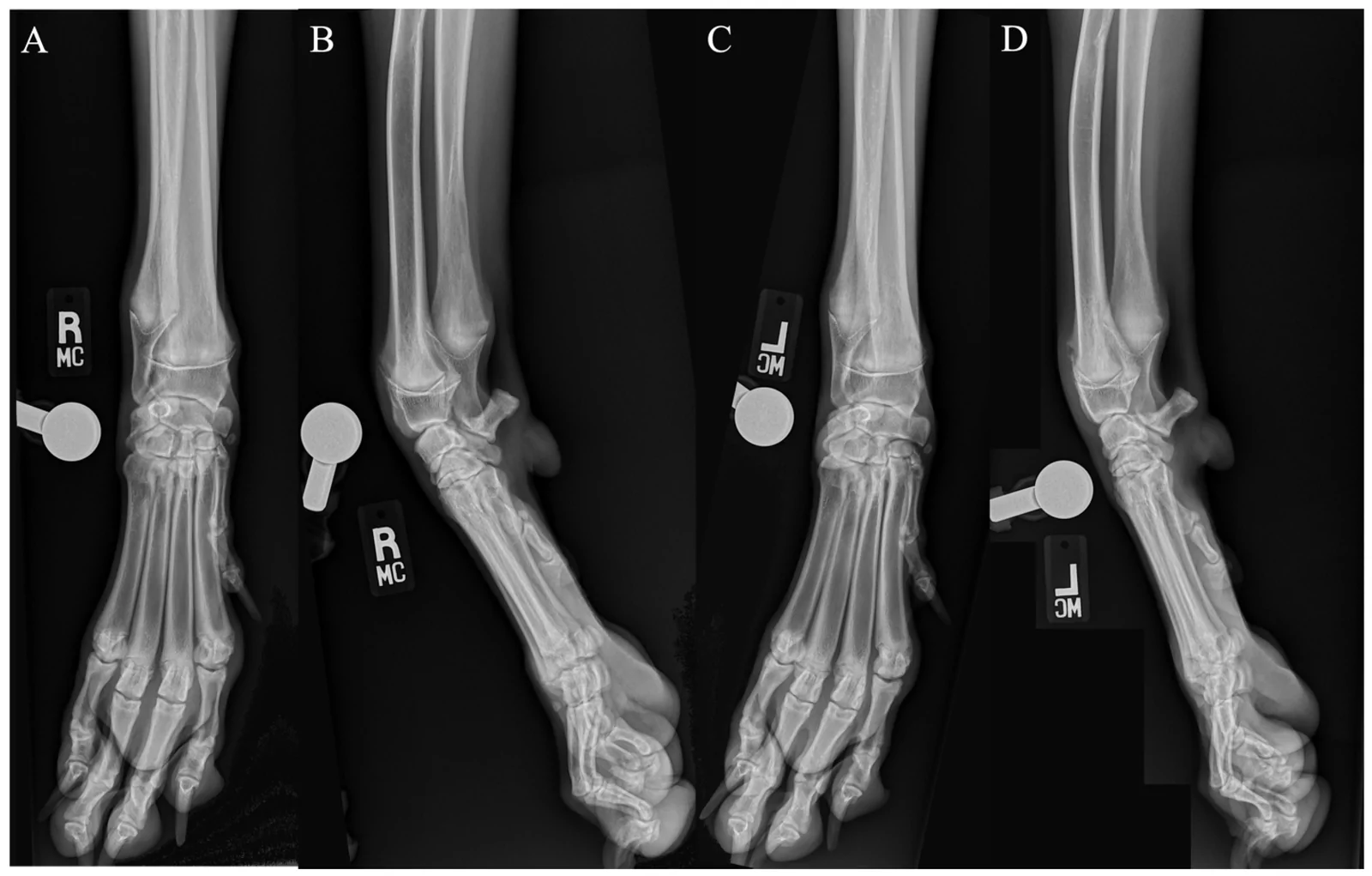
Orthogonal radiographs of the right (**A**,**B**) and left (**C**,**D**) distal antebrachium, carpus, and manus revealing bilateral valgus deformity of the distal portion of metacarpals II–V with thickening of the lateral cortex of these bones distally. Both second metacarpal bones have an apparent mid-diaphyseal varus deformity on the craniocaudal projections. There is mild heterogeneity of the distal radial and ulnar metaphyseal areas, as well as visible cutback zones, which may represent normal findings for a young skeletally immature giant breed dog. There is a poorly defined vertical flame-shaped radiolucency surrounded by sclerosis in the distal right ulnar metaphysis, suggestive of cartilage core retention.

Bilateral orthogonal radiographs of the antebrachium, carpus, and manus were acquired. Visual assessment of the radiographs revealed a bilateral valgus deformity of the distal portion of metacarpal bones II–V with thickening of the lateral cortex of the distal third of these bones. Both second metacarpal bones had an apparent mid-diaphyseal varus deformity on craniocaudal projections, and there was also valgus angulation centered in the distal metaphyseal region. There was no appreciable sagittal plane deformity. There was mild heterogeneity of the distal radial and ulnar metaphyseal regions, as well as visible cortical cutback zones—these findings were considered normal for a young skeletally immature giant breed dog. In addition, there was a poorly defined, vertical, flame-shaped radiolucency surrounded by sclerosis in the central region of the distal right ulnar metaphysis, suggestive of cartilage core retention (Figure 2). Neither antebrachium had overt frontal plane deformity, with a left anatomic medial proximal radial angle (aMPRA) of 83° and anatomic lateral distal radial angle (aLDRA) of 84° and right aMPRA of 82° and aLDRA of 84° [8,9].

**Figure 3:**
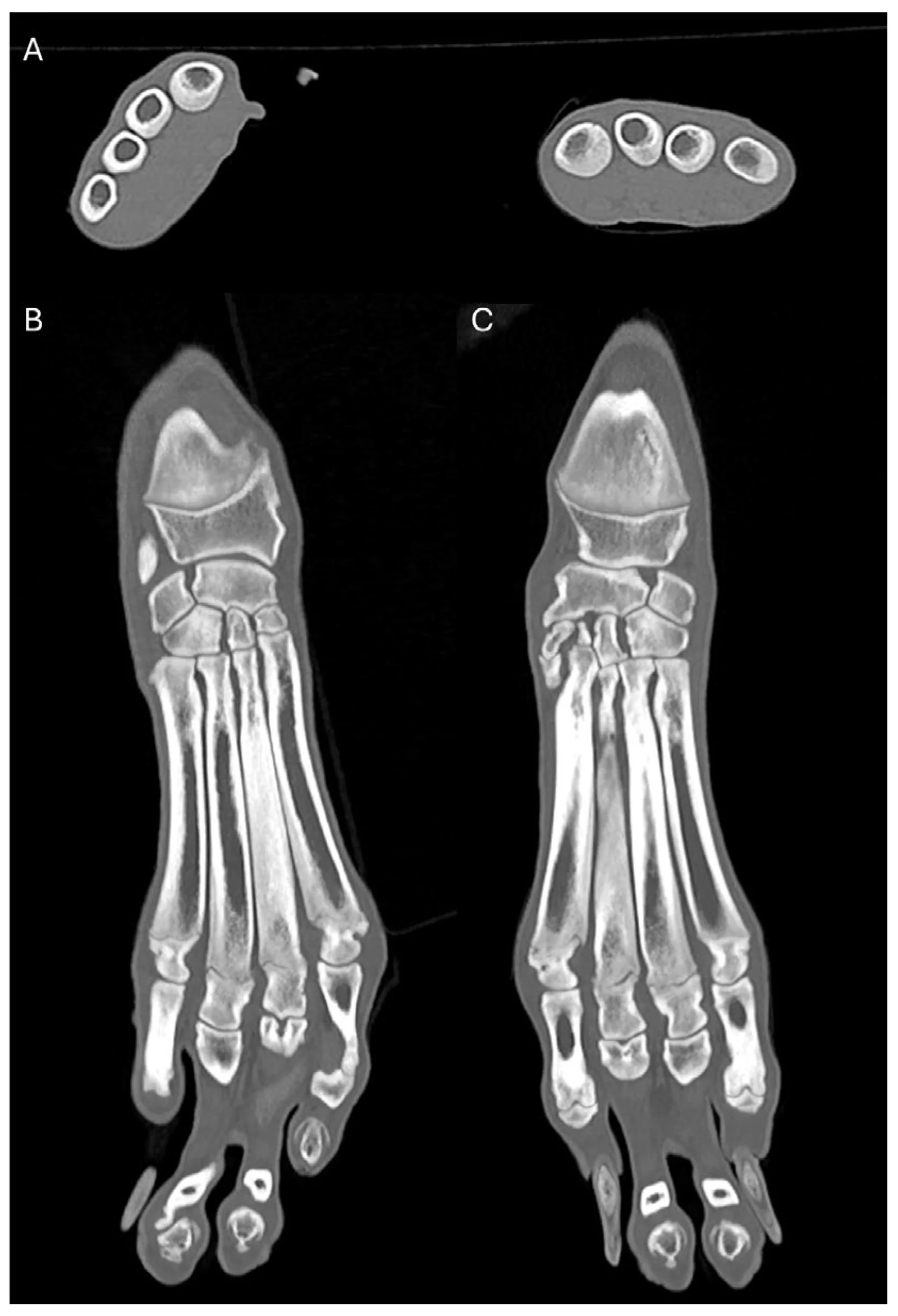
CT transverse image in a bone algorithm at the level of the metacarpal bones of both manus (**A**), as well as dorsal reformats in a bone algorithm of the right (**B**) and left (**C**) manus. In (**A**), a solid, well-defined, smoothly marginated, and continuous periosteal reaction is visible along the palmarolateral aspect of metacarpals II–IV bilaterally. In (**B**,**C**), the distal physes of the metacarpals II–V are asymmetrical, with the medial aspect of the physes closed or near closed, with greater physeal width laterally.

Preoperative CT of both manus and the distal antebrachia was performed using a 160-slice multidetector row, helical CT unit (Aquilion Prime, Toshiba Medical Systems, Inc., Tustin, CA, USA). Subjective evaluation of the CT revealed a bilateral distal physeal asymmetry of metacarpal bones II–V, with narrowing of the medial aspect of the distal physes and greater physeal width laterally, leading to bilateral distal metaphyseal valgus deformity. Additionally, there was an apparent bilateral mid-diaphyseal varus deformity of metacarpal bone II; however, when metacarpal bone II was rotated about its longitudinal axis and individually viewed in the frontal plane using a 3D reconstruction, the varus deformity was nominal. There was lateral cortical thickening affecting metacarpal bones II–V bilaterally. A solid, well-defined, smoothly marginated, and continuous periosteal reaction was also noted surrounding the palmarolateral aspect of metacarpals II–V bilaterally. The distal ulnar and radial metaphyses were moderately heterogenous with hypo- and hyperattenuating regions bilaterally (Figure 3).

### 2.3. Three-Dimensional Virtual Surgical Planning

**Figure 4:**
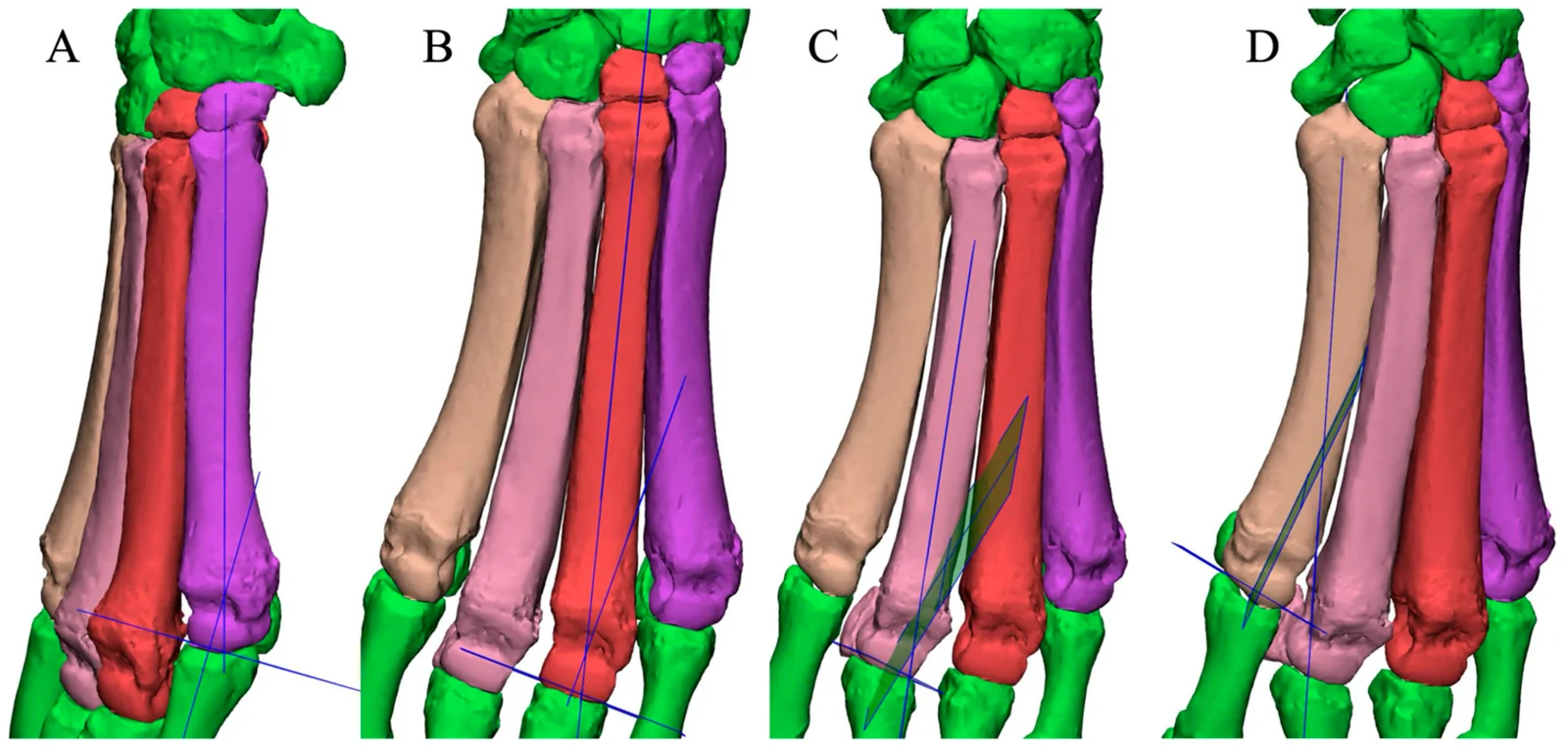
Individual 3D reconstructed frontal plane images of right metacarpal bones II (**A**), III (**B**), IV (**C**) and V (**D**) used to establish the longitudinal location and magnitude of each deformed metacarpal bone’s center of rotation of angulation (CORA). The manus was rotated about the longitude axis to obtain frontal plane images of the individual metacarpal bones. Planes were established representing each bone’s proximal and distal anatomic axes, with the intersection of those axes defining the neutral CORA. The linear distance (in mm) from the distal articular surface of the metacarpal bone to the CORA was measured along the distal anatomic axis to define the longitudinal location of the CORA, and the acute angle of intersection between the two anatomic axes defined the magnitude of the CORA.

A 3D reconstruction and VSP were conducted using an image processing software program (Mimics ver. 25.0, Materialize NV, Leuven, Belgium) and a biomodelling software program (3-matic 18.0, Materialize NV, Leuven, Belgium), respectively. Definition of the deformity as well as development of the VSP were subjective [10]. Both manus were rotated around the longitudinal axes to obtain frontal plane images of each individual metacarpal bone, which were then used to establish proximal and distal anatomic axes for each deformed metacarpal. A longitudinal plane that effectively bisected the medial and lateral cortices of each metacarpal bone’s proximal segment defined the proximal anatomic axes. A second longitudinal plane that passed through the mid-point of the distal articular surface of each metacarpal bone and was oriented perpendicular to a line drawn between points on the distal abaxial margins of the distal articular surface of each metacarpal bone defined the distal anatomic axes. The intersection of each metacarpal bone’s proximal and distal anatomic axes defined the neutral center of rotation of angulation (CORA) (Figure 4; Table 1). Overt sagittal and torsional deformity of the metacarpal bones were not apparent.

**Figure 5:**
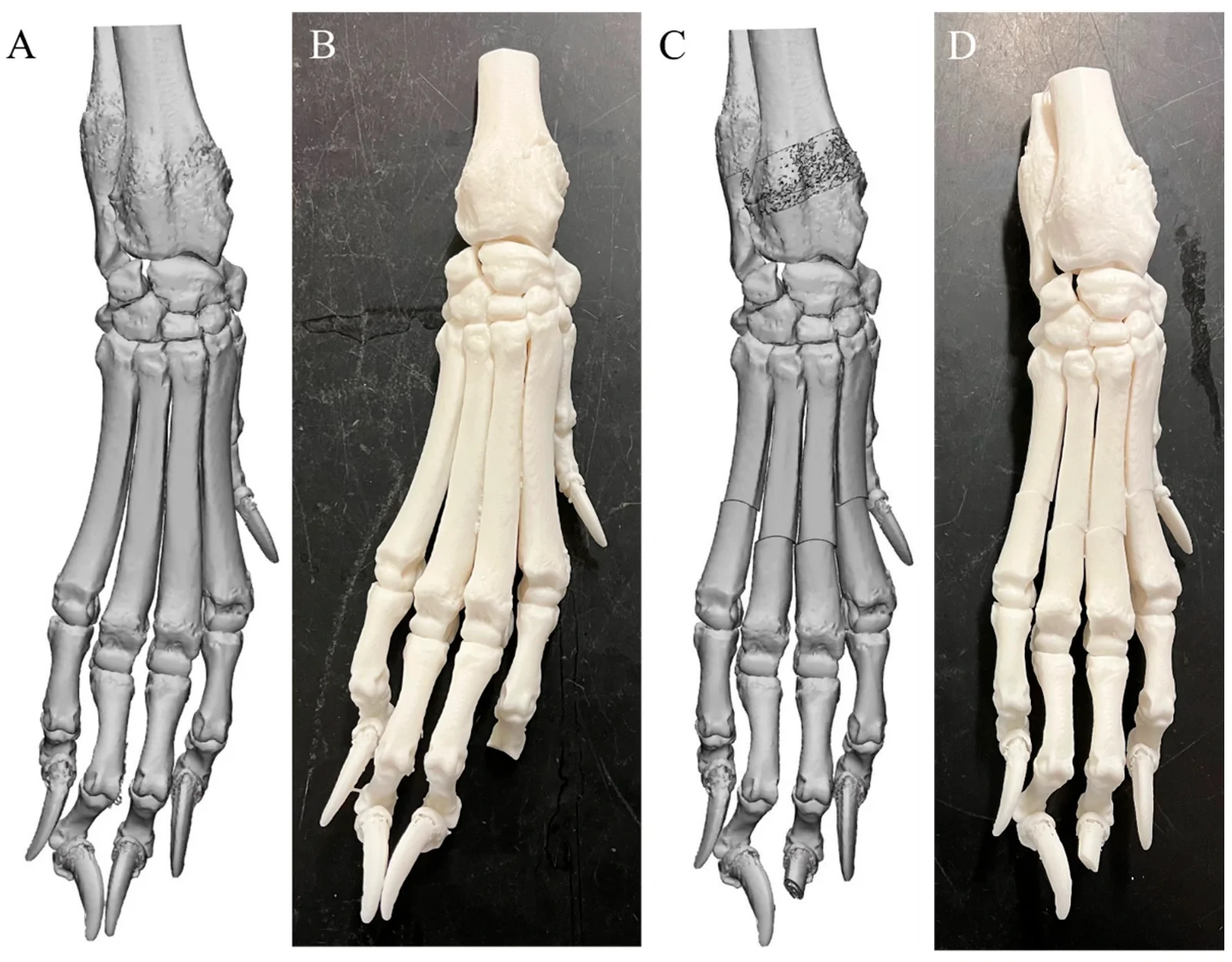
Preoperative and postoperative virtual correction images and models of the right thoracic limb. Reconstructed image (**A**) and three-dimensional (3D) printed model (**B**) of the deformed right manus. Reconstructed image after virtual correction of the deformity (**C**), simulating the planned postoperative alignment. Physical 3D-printed model of the virtually corrected manus (**D**).

Because of the distal location of the CORA in metacarpal bones II–IV, and to allow sufficient length of each distal metacarpal bone segment to accommodate plating while avoiding impedance between adjoining metacarpal bones during realignment, correction was planned by performing a 10 mm radial-shaped osteotomy positioned 40 mm proximal to the distal articular surface of each metacarpal bone. The convex surface of the blade was positioned facing proximally, with the concave surface facing distally. Each metacarpal bone was rotated about its longitudinal axis until viewed in the frontal plane, and a 10 mm radius circle was positioned and centered 30 mm proximal to the distal articular surface of metacarpal bones II–V. The arc of each superimposed circle represented the location of the proposed osteotomy, and each metacarpal bone was segmented into proximal and distal segments. The resultant distal metacarpal segments were all 40 mm in length, ensuring that there would be an adequate segment of bone to accommodate a 1.9–2.5 mm linear plate and three screws to stabilize each distal metacarpal segment. Each metacarpal bone was virtually segmented, from dorsal-to-palmar, through the arc, and the distal segment was rotated medially in the frontal plane until the deformity was visually corrected and the distal metacarpal segments of metacarpal bones III and IV and the phalanges of digits II–V were oriented parallel with a line drawn bisecting the adjacent cortices of the proximal segments of metacarpal bones III and IV in the frontal plane. The virtual correction was initially performed on the second metacarpal bone and sequentially performed, from medial-to-lateral, on the third, fourth, and fifth metacarpal bones. The deformed manus and the corrected manus bone models were 3D-printed (Form 3BL; Formlabs Inc., Somerville, MA, USA) using biocompatible resin (BioMed White Resin, Formlabs, Somerville, MA, USA) (Figure 5). The models were sterilized using ethylene oxide and utilized during surgery for visual corroboration of execution of VSP and to serve as a template for plate contouring.

### 2.4. Surgical Correction

Surgery of the right and left manus was staged and performed nine weeks apart. Corrective surgery of the right manus was performed first, as the dog was clinically more affected on that limb. The dog was premedicated for anesthesia with methadone 0.3 mg/kg IV and dexmedetomidine 5 mcg/kg IV. General anesthesia was induced with propofol 1 mg/kg IV and ketamine 1 mg/kg IV and maintained using isoflurane in oxygen. A radial, ulnar, median, and musculocutaneous nerve block was performed on the right thoracic limb using dexmedetomidine 0.1 mcg/kg and bupivacaine (0.5%) 1.3 mg/kg [11]. Cefazolin was administered at 22 mg/kg IV 30 min prior to starting surgery and every 90 min thereafter. The right thoracic limb was clipped, including all interdigital hair, and aseptically prepared for surgery.

The dog was positioned in dorsal recumbency, and a medial skin incision was made extending from the proximal diaphysis of the second metacarpal bone to the bone’s distal articulation with the first phalanx [12]. A caliper was used to measure a point 40 mm from the metacarpophalangeal articulation to the planned location of the radial-shaped osteotomy on the dorsal cortex of the second metacarpal bone. This location was marked with a 0.9 mm Kirschner wire, and a dorsal-to-palmar radial-shaped osteotomy was performed at this location with a 10 mm radial saw blade (De Puy Synthes, Warsaw, IN, USA). A six-hole, 1.9–2.5 mm locking, straight plate (Straight Support, Fixin Intrauma, Rivoli, Italy) was contoured just prior to application to the medial surface of the second metacarpal bone on the corrected bone model. The position of the distal segment was rotated medially in the frontal plane until the displacement of the two bone segments at the osteotomy was visually similar to the corrected bone model, and alignment of the digit was deemed appropriate. The contoured plate was positioned on the medial aspect of the second metacarpal with the plate in direct contact with the medial cortex of both the proximal and distal metacarpal bone segments. The plate was applied with six 2.5 mm locking screws (Fixin Intrauma, Rivoli, Italy). A dorsal approach was performed between metacarpal bones III and IV extending from the base of the metacarpal bones to the distal articulations with the first phalanx [12]. The extensor tendons and the associated neurovascular bundle were carefully elevated from the third and fourth metacarpal bones and were preserved. Radial-shaped osteotomies were performed on the dorsal surface of the third and then fourth metacarpal bones in the same manner as previously described; however, due to the more distal location of the CORA of the third metacarpal bone, a decision was made intra-operatively to perform the osteotomy 25 mm proximal to the metacarpophalangeal joint, and place an intramedullary pin, rather than a plate, to stabilize the osteotomy. A 2.4 mm Steinmann pin (IMEX Veterinary, Inc., Longview, TX, USA) was placed in retrograde fashion in the distal osteotomy segment, exiting through the dorsal cortex of the head of the third metacarpal bone. The distal metacarpal segment was then rotated medially until visual alignment of the digit appeared appropriate, and the pin was advanced into the proximal metacarpal segment and seated in the metaphysis. The pin was cut short with about 4 mm protruding from the head of the metacarpal bone. A dorsal-to-palmar radial-shaped osteotomy was made at the planned location in the fourth metacarpal bone, and the distal metacarpal segment was rotated medially in the frontal plane until visual alignment of the digit was deemed appropriate. A six-hole, 1.9–2.5 mm locking, straight plate (Straight Support, Fixin Intrauma, Rivoli, Italy), contoured to the dorsal surface of the fourth metacarpal bone on the corrected bone model, was applied using six 2.5 mm locking screws. Lastly, a lateral approach was performed, exposing the length of the fifth metacarpal bone [12]. A dorsal-to-palmar radial-shaped osteotomy was performed in the same fashion as previously described for the second metacarpal bone. A six-hole, 1.9–2.5 mm locking, straight plate (Straight Support, Fixin Intrauma, Rivoli, Italy) was contoured to the lateral surface of the fifth metacarpal bone on the corrected bone model. The distal osteotomy segment was rotated medially in the frontal plane until the lateral cortex of the segment contacted the plate and visual alignment of the digit was deemed appropriate. The plate was applied with five 2.5 mm locking screws and one 1.9 mm locking screw. Each surgical site was copiously lavaged with sterile saline. Cancellous bone graft, harvested from the greater tubercle of the ipsilateral humerus, was placed at each osteotomy site. Closure of all incisions was routine.

Nine weeks following correction of the right manus, surgery was performed on the left manus using the printed model of the corrected left manus. The second procedure was performed similarly to the first, with two variations. First, two incisions were utilized rather than three; a lateral approach to metacarpal bone II was done as previously described, but a dorsal approach centered over metacarpal bone IV was used to access metacarpal bones III–V. During the approach, the dorsal common digital vein overlying the fourth metacarpal bone was inadvertently incised, and electrocautery was used to control repeated hemorrhage from the vein. Second, all metacarpal osteotomies were made at the location according to the VSP, and all were stabilized using six-hole, 1.9–2.5 mm locking, straight plates (Straight Support, Fixin Intrauma, Rivoli, Italy) and 1.9 mm or 2.5 mm locking screws (Fixin Intrauma, Rivoli, Italy).

### 2.5. Postoperative Management

After both surgeries, the dog was administered cefazolin 22 mg/kg IV q8h for 24 h and then transitioned to cephalexin 30 mg/kg PO q8h for 10 days, methadone 0.1 mg/kg IV q6h as needed, carprofen 2.2 mg/kg PO q12h for 14 days, gabapentin 13 mg/kg PO q8h for 30 days, trazodone 5 mg/kg PO q8h for 60 days, and acepromazine 1 mg/kg PO q8h for 60 days. The paw was placed in a palmar splint which extended proximally to the mid antebrachium.

The splint was changed the day after the initial surgery. The right manus had nominal swelling, with only minimal inflammation of the incisions. The splint was reapplied, and the dog was discharged three days following surgery. The splint was changed weekly and maintained for one month.

When the splint was removed the day following the second surgery, there was ecchymosis and moderate swelling of the skin and soft tissues on the dorsum of the left manus. The splint was reapplied and changed daily for the next three days. The swelling and ecchymosis became progressively worse each day. The dog was discharged with the paw in a splint, four days after surgery. The dog returned for a splint change one week after surgery and the appearance of the skin overlying the left manus had not improved. Fluorescent light therapy (Phovia, Klox Technologies, Laval, QC, Canada) was initiated and performed during splint changes on days seven-, nine-, 14-, and 23 post-operatively. The bruising and swelling had completely resolved at the time of the final treatment, and splinting was discontinued at four weeks following surgery.

### 2.6. Outcome

**Figure 6:**
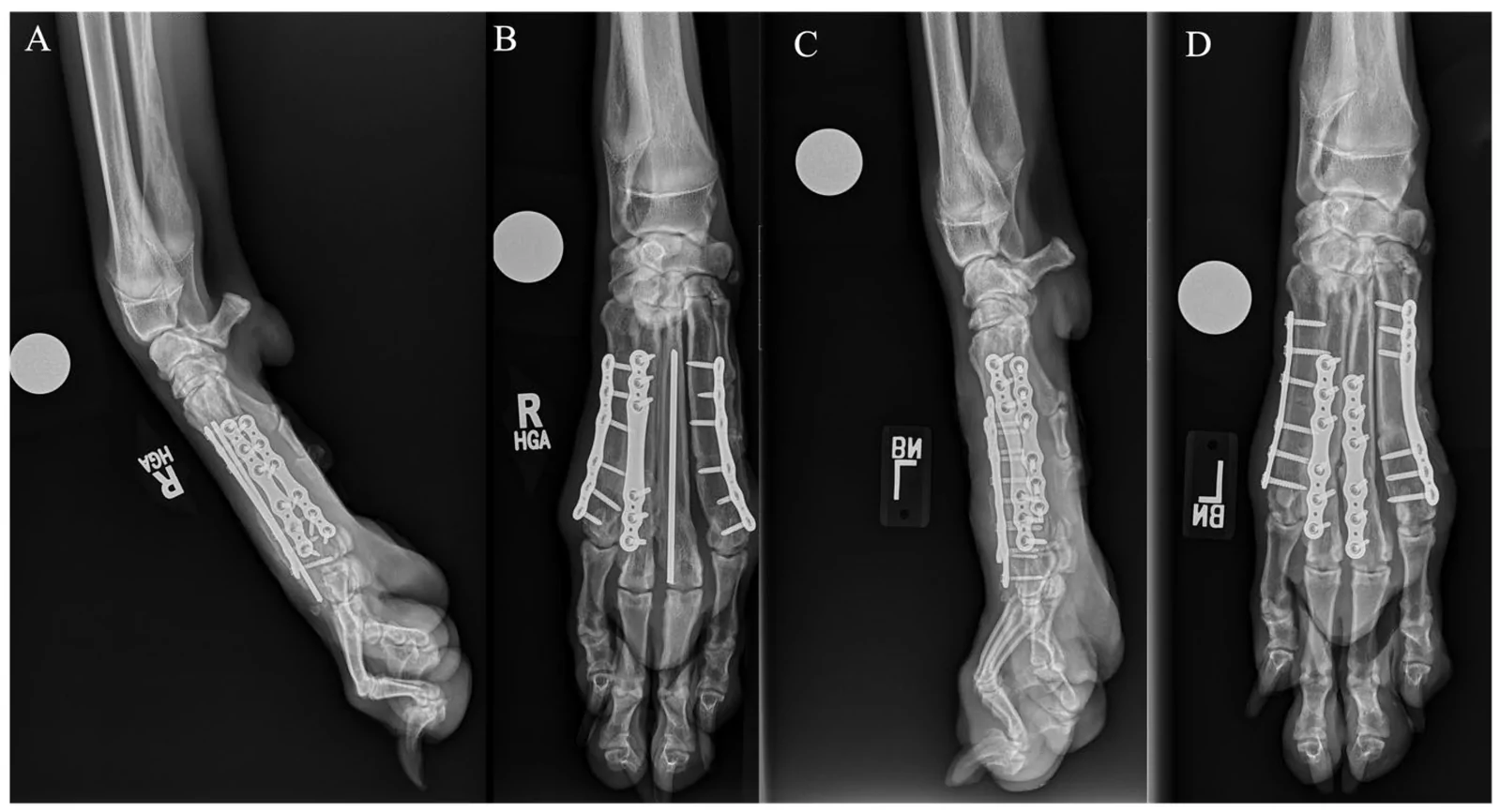
Orthogonal radiographs of the right (**A**,**B**) and left (**C**,**D**) manus obtained eight and four weeks following surgery, respectively. Radiographs of both manus revealed complete osseous union of the osteotomies. No complications related to the implants were appreciated. The distal portion of metacarpal bones III and IV and phalanges of digits II–V are aligned more parallel with a line bisecting the adjacent cortices of the proximal segments of metacarpal bones III and IV in the frontal plane, with resolution of the prior distal metacarpal valgus.

The dog returned for re-evaluation four weeks following correction of the right manus. At this time, the owners reported the dog was doing well at home, consistently bearing weight on the splinted right thoracic limb, and they had no concerns. On orthopedic examination, the dog was placing substantial weight on the splinted right thoracic limb but still had postural and gait abnormalities of the left thoracic limb. The splint was removed, and the right manus no longer had valgus angulation and was visually aligned such that the distal segments of metacarpal bones III and IV as well as the phalanges of digits II–V were oriented parallel with a line which bisected the proximocentral longitudinal axis of the metacarpus in the frontal plane. The dog was not painful on palpation of the right metacarpus. Radiographs of the right manus revealed substantial new bone formation partially bridging the metacarpal osteotomies. Eight weeks post-operatively, the dog did not have an appreciable right thoracic limb lameness, and radiographs revealed complete osseous union of the osteotomies. Complications associated with the implants were not appreciated (Figure 6A,B).

When the dog returned for re-evaluation four weeks following correction of the left manus, the owners noted that the dog appeared to be stiff in both thoracic limbs when initially rising, but otherwise the dog was ambulating well without appreciable lameness. On orthopedic examination, the dog was placing substantial weight on the splinted left thoracic limb. The splint was removed, the left manus was visually appropriately aligned, and the dog was not painful on palpation of the left manus. Radiographs of the left manus revealed osseous union of the osteotomies, and there were no complications associated with the implants (Figure 6C,D).

**Figure 7:**
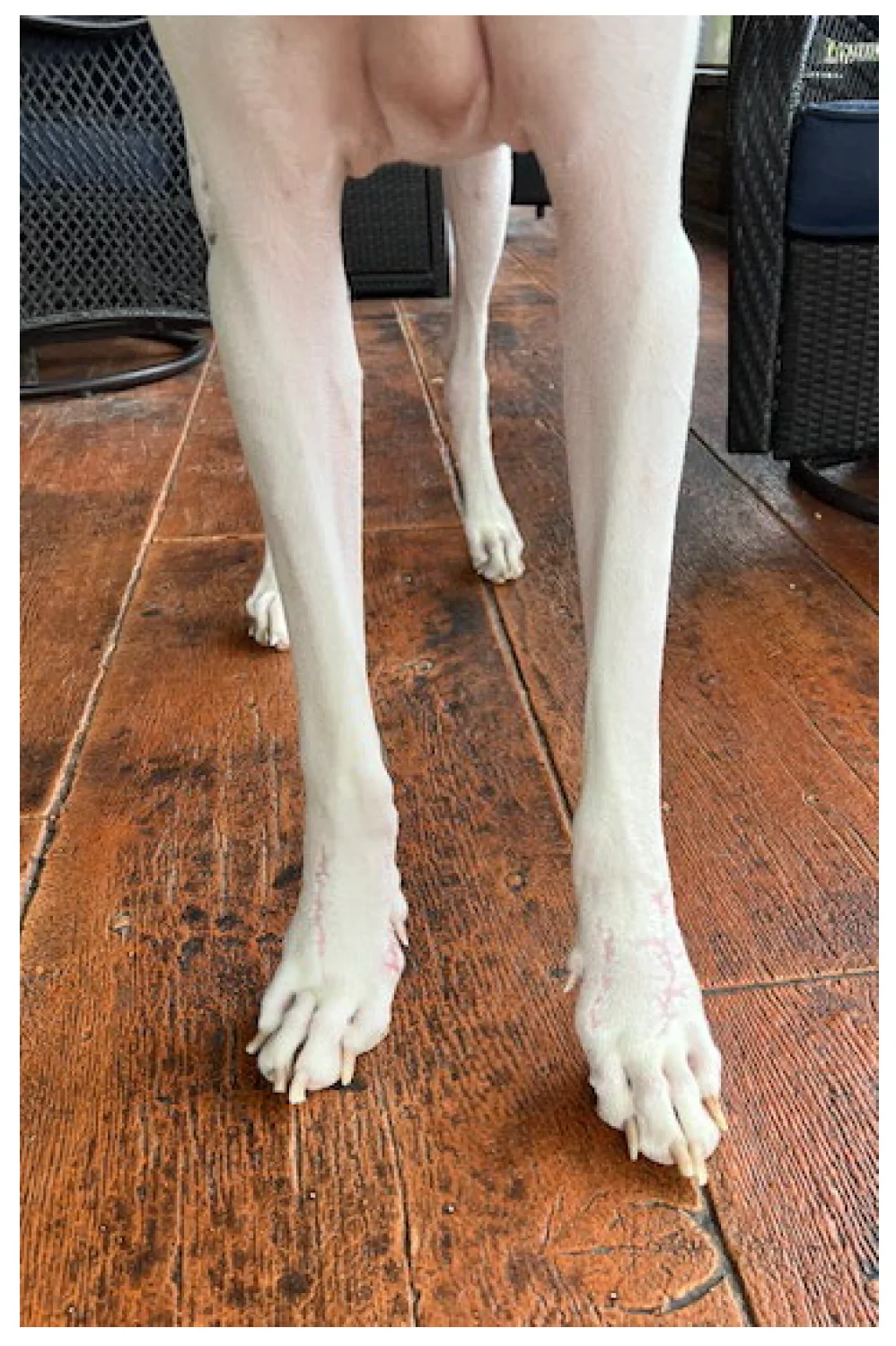
Image of the dog’s thoracic limbs 79 weeks following the initial surgery. The marked bilateral valgus angulation of both manus noted prior to surgery has been markedly visually improved; however, there is still some residual valgus angulation of the phalanges, most notably in the right thoracic limb.

Radiographs of both manus were obtained by the referring veterinarian 13 and 22 weeks following correction of the left and right manus, respectively. The radiographs revealed progressive remodeling of the healed metacarpal bones, and no complications related to the implants. At that time, the owners reported that the dog was ambulating well without lameness except for occasional thoracic limb stiffness when first rising after periods of prolonged recumbency. All medications had been previously discontinued, and the dog had resumed normal unrestricted activity. The dog was examined 70 and 79 weeks following correction of the left and right manus, respectively. The dog ambulated without appreciable lameness, and the owners were highly satisfied with the results of the surgeries. The valgus angulation of both manus was visually markedly improved, but the dog intermittently postured with both manus in slight valgus, with valgus angulation being slightly more pronounced in the right manus. The predominance of the residual valgus deviation in both manus seemed to involve the phalanges rather than the metacarpus, although demonstratable laxity of the metacarpophalangeal and interphalangeal joints was not appreciated on palpation (Figure 7).

## 3. Discussion

Metacarpal deformity has been reported in dogs secondary to fracture malunion [6]. The deformity in the dog in this report presumably was developmental, as the deformity was bilateral, and the onset of lameness and subsequent deformity were insidious, progressive, and noted before the dog had reached skeletal maturity. There was no known history of trauma, and abnormalities consistent with prior trauma were not evident on the preoperative radiographs or CT.

The CT abnormalities suggest premature bilateral asymmetric closure of the distal metacarpal physes, which may have ultimately led to valgus deformity of the distal metacarpal bones. We can only speculate that the physeal abnormalities resulted from abnormal thoracic limb posturing, which exerted excessive compressive loads on the medial portion of the growth plate perpetuating dysplastic valgus deformity. Valgus posture would exert great compressive loads on the medial portion of the distal metacarpal physes and, in accordance with the Hueter–Volkmann law, would retard physeal growth in this region [13]. This is consistent with the narrow medial physeal morphology appreciated on CT in this dog. An additional confounding observation is that retarded medial physeal growth with continued growth from the lateral physis would be expected to produce varus angulation, suggesting that medial physeal narrowing was most likely due to increased loading of that portion of the physis as the dog approached skeletal maturity. What precipitated the dog’s valgus posture is unknown, as there was no obvious antebrachial deformity, and the dog’s radial frontal plane joint reference angles were within normal range [8,9]. However, reassessment of the bone models suggests that there might have been some unappreciated torsional or rotational deformity to the distal thoracic limb that may have contributed to the development of metacarpal bone deformity. The CT of the manus only included the distal radius and ulna, limiting our ability to accurately reassess if torsional or rotational deformity of the antebrachia and/or the carpal articulations were a contributing factor. The periosteal reaction affecting multiple metacarpal bones bilaterally, as well as the bilateral distal radial and ulnar metaphyseal heterogeneity, might suggest prior or evolving hypertrophic osteodystrophy or metaphyseal osteopathy [14,15]. However, the periosteal reaction could also be secondary to abnormal weight-bearing and excessive stress on the lateral aspect of the metacarpal bones.

The application of VSP and 3D-printing in veterinary orthopedics has provided surgeons with powerful technology for addressing complex orthopedic deformities [11,12,13]. Virtual frontal plane realignment of the segmented metacarpal bones was subjective, with alignment of the distal portion of metacarpal bones III–IV and the phalanges of digits II–V parallel with a line drawn that bisected the adjacent cortices of the proximal segments of metacarpal bones III and IV being the primary objective [10]. Virtual surgical planning and the use of printed models is purported to enhance intraoperative accuracy and efficiency, increasing surgeon confidence and reducing surgical time [10,16,17,18,19,20,21]. In the case described in this report, the dog’s CT data was used to develop the virtual surgical plan and to generate 1:1 scale, 3D-printed anatomical models of the deformed and corrected manus. These physical replicas afforded a tangible, 3D understanding of the intricate multi-bone deformity that could not be fully appreciated from on-screen digital imaging alone. The models provided a comprehensive ex vivo representation of the intended surgical outcome, corroborated the location of the planned radial-shaped osteotomies and realignment of each individual metacarpal bone before entering the operating room, and facilitated proper contouring of bone plates during surgery. While 3D-printing can also be used to design and fabricate patient-specific surgical guides, we choose not to use osteotomy and/or reduction guides because we felt guides would be impractical in this particular case due to the limited surgical exposure afforded to individual metacarpal bones, interference of adjacent metacarpal bones in allowing guide placement, and the small, cylindrical morphology of the metacarpal bones that would have prevented stable and accurate guide placement [18,19].

Reported osteotomy techniques for correcting metacarpal deformities secondary to fracture malunion in human patients include closing and opening wedge ostectomies, step-cut rotational osteotomy, and trapezoid rotational bone graft osteotomy [7,22,23]. While outcomes are generally considered good following surgical correction of metacarpal deformities in people, small deviations in the measured osteotomy site can result in complications such as persistent deformity, as well as delayed or non-union [22,23]. We were unable to locate prior analogous reports describing surgical correction of metacarpal deformities in dogs. Radial-shaped osteotomies were employed in the current case due to the small size of the metacarpal bones and the distal location of the CORA in most of the metacarpal bones. Radial-shaped osteotomies allow the osteotomies to be positioned remote to the CORA, which is particularly advantageous when the CORA is positioned adjacent to a joint. The central axis of the radial cutting blade can be positioned at the neutral CORA which allows the osteotomy to be positioned more proximal, resulting in a longer juxta-articular bone segment, making plate stabilization feasible [8,24]. In the current case, the fixed point of the circle of the proposed osteotomy was still located 9 to 17 mm proximal to the CORA in five of the metacarpal bones stabilized with a plate. Performing radial-shaped osteotomies helped to mitigate invoking a secondary translational deformity of the distal metacarpal bone segments, resulting in near-collinearity of the proximal and distal anatomic axes of third and fourth metacarpal bones. We elected not to employ an opening wedge osteotomy or closing wedge ostectomy in the case presented here, as performing either of those osteotomies remote enough from the CORA to allow sufficient space for plate placement would have invoked a more profound secondary translational deformity [8,24]. One negative consequence of using a radial-shaped osteotomy is that the ability to perform compensatory translation through the osteotomy is limited [5]. Fortunately, the degree of an induced secondary deformity in any of the metacarpal bones was considered nominal. The metacarpal deformities in the dog in the current report were primarily confined to the frontal plane, which permitted the use of radial-shaped osteotomies. If the deformities had been multiplanar or had a substantial torsional component, alternative osteotomy techniques may have been more appropriate [25].

Plates were used to stabilize all but one of the osteotomies. Plate fixation was utilized because of the dog’s large size and the need to correct all four of the weight-bearing metacarpal bones in both manus, and we anticipated substantial loading of the realigned metacarpal bones during the postoperative convalescent period. An intramedullary pin was used instead of a plate to stabilize the right third metacarpal bone to allow positioning the osteotomy more distal, closer to the CORA, of this metacarpal bone. The CORA of metacarpal III was located more distal, 8.4 mm from the metacarpophalangeal joint, than in the other metacarpal bones. No complications occurred as a result of pinning this single metacarpal bone; however, the authors do not believe that intramedullary pinning would have been sufficient as the sole method of fixation of all the metacarpal bones in this 48 kg dog.

Following correction of the left manus, the dog developed substantial and progressive edema and ecchymosis surrounding the dorsal incision which was suspected to be due to damage to the dorsal common digital vein during the approach. Electrocautery was used to control repeated hemorrhage from a small perforation in the vein which likely caused thrombosis of the vessel. A single skin incision was used to approach the III–V metacarpal bones of the left manus, and that incision was made directly over the fourth metacarpal bone predisposing the dorsal common digital neurovascular bundle to injury. Ecchymosis and edema may have been aggravated by continued hemorrhage and obstructed venous return during the immediate postoperative period, resulting in increased tension on the surgical site, producing a tourniquet effect affecting the metacarpus and digits. During surgery on the right manus, a dorsal skin incision was made between the third and fourth metacarpal bones, and a separate lateral approach was made to the fifth metacarpal, which would appear to mitigate the potential for iatrogenic vascular injury.

Fluorescence biomodulation therapy was used on the left manus to help encourage wound healing and combat possible ischemic necrosis of the skin. This therapeutic modality is a form of light therapy utilizing low-energy photons of varying wavelengths. These photons have a variety of beneficial effects within a wound bed, including reducing inflammation, increasing keratinocyte proliferation, improving perfusion, promoting collagen synthesis, and stimulating angiogenesis [25]. A previous study evaluating the use of fluorescence biomodulation therapy for surgical wound healing in dogs reported improved histologic scores, characterized by reduced inflammation of the dermis, greater and more regular deposition of collagen, and complete re-epithelialization, for wounds that received fluorescent therapy compared to untreated surgical wounds [26]. The macroscopic appearance of the left manus surgical site progressively improved, as swelling and bruising regressed between each fluorescent treatment, although the observed chronologic improvement may have occurred regardless of fluorescent therapy.

There are numerous limitations to this case report. This is an isolated case involving a single dog affected with unique morphologic anomalies. The authors can only speculate that the etiology was developmental, given that lameness and conformational changes were initially noted prior to skeletal maturity and progressed prior to presentation. Definition of each metacarpal bone’s deformity was admittedly subjective, as a best-fit bisecting line was established to define both proximal and distal anatomic axes as well as the CORA of individual metacarpal bones; however, the authors were unable to find a published study performed to establish joint reference lines and anatomic or mechanical axes in dogs that could be applied to this case. Using CT data appears to be more effective than radiographs in performing this process, particularly for the abaxial metacarpal bones II and V, due to the arched arrangement of the bones in the distal portion of metacarpus. Virtual surgical planning was also performed subjectively, with the primary goal being to realign the distal portion of the metacarpal bone and the phalanges parallel with the frontal plane centroproximal metacarpal longitudinal mechanical axis. However, similar methodology has been used to effectively define and plan antebrachial deformity corrections in dogs [10]. Obtaining postoperative CT scans in this dog would have allowed us to more accurately assess our proficiency in executing our virtual plan [10,16], but postoperative CTs were not obtained due to the owners’ financial constraints. Additional limitations include that case information was gathered retrospectively, and our clinical and radiographic evaluations were primarily subjective.

## 4. Conclusions

This report documents a presumed developmental valgus deformity of both manus which resulted in bilateral thoracic limb postural and gait abnormalities. Successful bilateral correction of metacarpal deformities was facilitated by VSP and the intra-operative use of 3D-printed models, resulting in an acceptable long-term functional outcome. The alignment of the metacarpal bones was substantially improved following the procedures, as was the dog’s posture and gait. The slight residual valgus deformity noted at the final evaluation may have been related to ligamentous laxity in the metacarpophalangeal and interphalangeal joints, although demonstrable laxity was not appreciated on palpation.

## Figures and Tables

**Table 1:** Longitudinal location (mm proximal to the distal articular surface) and magnitude (in degrees) of the center of rotation of angulation (CORA) of each of the dog’s deformed metacarpal bones.

CORA	Right Metacarpal Bones				Left Metacarpal Bones			
	II	III	IV	V	II	III	IV	V
Location (mm)	12.6	8.4	18.4	31.5	14.2	18.3	20.6	40.5
Magnitude	16.0°	14.1°	19.8°	21.4°	12.1°	7.3°	14.0°	14.7°

## Data Availability

The data presented in this study are available on request from the corresponding author due to client and patient privacy.

## Abbreviations

The following abbreviations are used in this manuscript:

VSP	Virtual surgical planning
3D	Three-dimensional
CORA	Center of rotation and angulation
aMPRA	Anatomic medial proximal radial angle
aLDRA	Anatomic lateral distal radial angle
